# Lumican Is Overexpressed in Lung Adenocarcinoma Pleural Effusions

**DOI:** 10.1371/journal.pone.0126458

**Published:** 2015-05-11

**Authors:** Rocco Cappellesso, Renato Millioni, Giorgio Arrigoni, Francesca Simonato, Brasilina Caroccia, Elisabetta Iori, Vincenza Guzzardo, Laura Ventura, Paolo Tessari, Ambrogio Fassina

**Affiliations:** 1 Department of Medicine, Surgical Pathology & Cytopathology Unit, University of Padua, Padua, PD, Italy; 2 Department of Medicine and Proteomics Center, University of Padua; Padua, PD, Italy; 3 Department of Biomedical Sciences, University of Padua and Proteomics Center of Padua University, Padua, PD, Italy; 4 Department of Medicine, Internal Medicine 4, University of Padua, Padua, PD, Italy; 5 Department of Medicine, Metabolism Division, University of Padua, Padua, PD, Italy; 6 Department of Statistical Sciences, University of Padua, Padua, PD, Italy; Memorial Sloan-Kettering Cancer Center, UNITED STATES

## Abstract

Adenocarcinoma (AdC) is the most common lung cancer subtype and is often associated with pleural effusion (PE). Its poor prognosis is attributable to diagnostic delay and lack of effective treatments and there is a pressing need in discovering new biomarkers for early diagnosis or targeted therapies. To date, little is known about lung AdC proteome. We investigated protein expression of lung AdC in PE using the isobaric Tags for Relative and Absolute Quantification (iTRAQ) approach to identify possible novel diagnostic/prognostic biomarkers. This provided the identification of 109 of lung AdC-related proteins. We further analyzed lumican, one of the overexpressed proteins, in 88 resected lung AdCs and in 23 malignant PE cell-blocks (13 lung AdCs and 10 non-lung cancers) using immunohistochemistry. In AdC surgical samples, lumican expression was low in cancer cells, whereas it was strong and diffuse in the stroma surrounding the tumor. However, lumican expression was not associated with tumor grade, stage, and vascular/pleural invasion. None of the lung cancer cell-blocks showed lumican immunoreaction, whereas those of all the other tumors were strongly positive. Finally, immunoblotting analysis showed lumican expression in both cell lysate and conditioned medium of a fibroblast culture but not in those of A549 lung cancer cell line. PE is a valid source of information for proteomic analysis without many of the restrictions of plasma. The high lumican levels characterizing AdC PEs are probably due to its release by the fibroblasts surrounding the tumor. Despite the role of lumican in lung AdC is still elusive, it could be of diagnostic value.

## Introduction

Lung cancer is the leading tumor worldwide for incidence and mortality and is classified according to histological type into two broad classes: the small-cell lung carcinoma and the more common non-small-cell lung carcinoma, further divided in adenocarcinoma (AdC), squamous cell carcinoma, and large cell carcinoma [1,2]. AdC alone accounts for about 40% of all lung cancers, it is usually located in periphery of the lung and may present with a pleural effusion (PE). Despite the recent advances in lung cancer treatment, prognosis is still poor, mainly because of diagnostic delay. Thus, it is crucial to identify new biomarkers involved in the molecular mechanisms underlying tumor genesis and progression, which could be useful for early detection or for the development of novel therapeutic strategies.

Proteomics provides an unbiased opportunity to discover new potential pathogenic mechanisms and therapeutic targets, as well as biomarkers of disease [3]. Such approaches are generally applied to a small cohort of patients to generate hypotheses that need to be validated in larger cohorts [4]. To date, proteomics has been rarely exploited to discover new aspects of AdC and the findings have never been properly validated [5]. The use of high-throughput technologies, such as shotgun proteomics, which combines liquid chromatography (LC) with mass spectrometry (MS), allows the identification and quantification of a large number of proteins even from small specimens (both solid tissue and liquid material). However, validation of the altered expression of such a large amount of proteins in biologic processes and in diseases is often difficult. Plasma is one of the most common body fluids used for the identification and validation of novel biomarkers of disease. To date, the plasma proteome associated with many diseases has been intensively investigated, although it is analytically challenging [6,7]. Indeed, the ten most abundant plasma proteins account for approximately 90% of the total protein content, whereas other proteins are present in a very wide dynamic range, spanning more than 10 orders of magnitude in terms of concentration [8]. Thus, the complexity of the plasma proteome exceeds the current analytical capacity of conventional approaches to isolate low abundance proteins that might be informative biomarkers. Malignant PE, instead, seems to be a valid alternative source for biomarker discovery. Indeed, the prolonged cancer cell growth in the closed pleural cavity concentrates the secreted proteins clearing many of technical challenges associated with the plasma proteome analysis; the emphasis is shifting from cancer cells to the non-cellular portion of PE.

In this study, we first used the isobaric Tags for Relative and Absolute Quantification (iTRAQ) approach as a gold-standard proteomic strategy to compare proteins present in PE of patients with and without AdC. Then we selected lumican as candidate target to be further validated in a series of surgical and cytological AdC samples.

## Materials and Methods

### Materials

This study was approved (No. 46728) by the Institutional Ethical Review Board of Padua University that waived the need for consent. The institute’s ethical regulations on research conducted on human tissues were followed. All fresh PE submitted for routine diagnostic evaluation to our Cytopathology Unit during 2010 were immediately centrifuged for 20 minutes at 2,000 x g to separate the cells from the supernatant fluid on their arrival at the laboratory. Cells were then processed to prepare the cytological smears, whereas the supernatant fluid was centrifuged again at 2,000 x g for 30 minutes to remove cellular debris and contaminants. Then, 10 ml of the fluid were transferred to a new container without disturbing the pellet and a tablet of cOmplete, Mini, EDTA-free protease inhibitor (Roche, Mannheim, Germany) was added. The mixture was gently shaken until complete dissolution of the tablet and readily stored at -80°C until further use. Among all these samples, we selected three PEs positive for AdC malignant cells at the cytological examination. All were obtained before the administration of chemotherapy and surgical treatment to the patients and were histologically confirmed. As controls, three PEs obtained from patients affected by congestive heart failure were also included; these samples were proven to be transudates according to Light’s criteria and were cytologically negative for neoplastic cells [9].

From the archives of the Surgical Pathology and Cytopathology Unit of the University of Padua for the period between 2010 and 2011, we retrieved the resected specimens of 88 lung AdCs (clinical and pathological data for these cases are reported in Table 1) and 5 normal lung tissue from distant areas in cancer pneumonectomies. In addition, 10 lung AdC, 3 NSCLC-not otherwise specified (NOS), 5 ductal breast carcinoma (no special type), and 5 serous ovarian carcinoma cell-blocks were also included. All the cases were reviewed and the diagnoses confirmed in all instances by 2 pathologists (AF and RC) according to the World Health Organization classification [2].

**Table 1:** Clinicopathological features of the lung adenocarcinoma series.

Variables	Numbers of patients	%
*Gender*		
Male	61	63.3
Female	27	36.7
*Vascular invasion*		
Present	40	45.5
Absent	48	54.5
*Pleural invasion*		
Present	32	36.4
Absent	56	63.6
*Grade*		
1	14	15.9
2	39	44.3
3	35	39.8
*TNM stage*		
I	25	28.4
II	25	28.4
III	26	29.5
IV	12	13.7

Patients classification according to gender, vascular/pleural invasion, grading and staging.

### Sample preparation for proteomic analysis

A small aliquot from each sample was diluted 10 times with MilliQ grade water for proteins quantification using microBCA (Thermo Fisher Scientific, Somerset, USA) method. Two pools were obtained by mixing equal quantities of proteins from AdC and non-neoplastic PE samples. Proteins (200 μg) from each pool were precipitated overnight at -20°C with four volumes of cold acetone. Protein pellets were dissolved in 5 ml of 50 mM ammonium bicarbonate, 0.1% Triton X-100 and sonicated on ice. Samples were centrifuged at 14,000 x g for 10 minutes at 4°C to remove insoluble debris. The supernatants were concentrated on 3 kDa molecular weight cut-off filters (Centricon Centrifugal Filter Devices with YM-3 membranes; EMD Millipore Corporation, Billerica, USA) followed by buffer exchange with 50 mM triethyl ammonium bicarbonate (TEAB). Protein content from each sample was quantified again using microBCA (Thermo Fisher Scientific) method.

### iTRAQ labeling

Proteins (100 μg) from each pool were diluted in iTRAQ dissolution buffer (TEAB 0.5 M) to a final volume of 100 μl. Samples were reduced, alkylated, and trypsin digested according to the iTRAQ kit manufacturer’s instructions. After digestion, 1 μg of each sample was used to confirm the digestion efficiency by LC-MS/MS. To reduce any potential variation introduced by the labeling reaction, each sample was split in two identical aliquots of 50 μg to perform two technical replicates with tag swapping. The labeling procedure was performed following the manufacturer’s protocol. To check the completeness of the labeling, 1 μg of each labeled sample was individually analyzed by LC-MS/MS. Acquired data were searched with Sequest search engine, setting the iTRAQ labeling as variable modification. Details about MS and data analyses are reported below. No unmodified peptides were identified and all peptides were correctly modified at the N-terminus and at each Lysine residue. Following iTRAQ labeling, the samples were pooled into one clean tube and vacuum concentrated in a SpeedVac system.

### Strong Cation Exchange Chromatography

The dried iTRAQ labeled sample was dissolved in 400 μl of an equilibration buffer composed by 5 mM KH_2_PO_4_, 25% acetonitrile, pH 2.9 (adjusting the pH with 1 M H_3_PO_4_). Strong cation exchange (SCX) was performed using a SCX cartridge (AB Sciex, Toronto, Canada). Elution of peptides was carried out in a step-wise manner, with 500 μl of the following concentrations of KCl in equilibration buffer: 50, 100, 150, 200, 350 mM. Each SCX fraction was then dried under vacuum, dissolved in 500 μl of 0.1% formic acid, and desalted using Sep-Pak C18 cartridges (Waters Associates, Milford, USA) according to the manufacturer’s instructions. Samples were finally dried under vacuum and stored at -20°C until the MS analyses were performed.

### Offgel fractionation

Peptides from iTRAQ pools were desalted using Sep-Pak C18 columns, eluted with 60% ACN and dried in a SpeedVac system. Focusing was performed on Agilent 3100 Offgel Fractionator (Agilent Technologies, Palo Alto, USA) according to the manufacturer’s protocol. After 1 h of rehydration with a voltage of 500 V, peptides were focused until 80 kVh was reached on 24 cm IPG strip (GE Healthcare, Milwaukee, USA) with a 4–7 pH range. The 24-well tray was used and the collected fractions were cleaned with Sep-Pak C18 columns, dried with a SpeedVac system and stored at -20°C until the MS analyses were performed.

### nLC-ESI-MS/MS Analyses

LC-MS/MS analyses were performed on a LTQ-Orbitrap XL mass spectrometer (Thermo Fisher Scientific) coupled online with a nano-HPLC Ultimate 3000 (Dionex, Sunnyvale, USA—Thermo Fisher Scientific). Samples from each SCX and IEF fraction were dissolved in 0.1% formic acid, obtaining a final concentration of about 1 μg/μl. To check the completeness of peptide labeling and for the analysis of the iTRAQ pools, 1 μg of each iTRAQ labeled sample before fractionation and of each SCX and IEF fraction was loaded onto a homemade 10 cm chromatographic column packed into a pico-frit (75 μm I.D., 10 μm tip, New Objectives) with C18 material (ReproSil, 300Å, 3 μm). Peptides were eluted with a linear gradient of acetonitrile/0.1% formic acid from 3% to 50% in 90 min at a flow rate of 250 nl/min. The instrument was programmed to perform a full scan at high resolution on the Orbitrap, followed by MS/MS scans on the three most intense ions acquired with CID fragmentation in the linear ion trap. On the same ions, a further HCD fragmentation was performed with detection on the Orbitrap, to record the low molecular weight ions necessary for the quantification [10]. To improve protein identification and quantification, data were searched with Mascot (as specified below) and all peptides identified with high or medium confidence were inserted into a static exclusion list. All samples were analyzed again under the same instrumental and chromatographic conditions.

### MS data analysis

Raw files from Orbitrap instrument were analyzed using Proteome Discoverer 1.2 (Thermo Fisher Scientific) connected to a Mascot server (Matrix Science, London, UK) and to Sequest Search Engine (Thermo Fisher Sicentific). Search was done against the human section of the Uniprot database (version 2012/07/26). Peptide tolerance was set to 10 ppm for MS spectra, fragment tolerance was set to 0.6 Da for MS/MS spectra. Enzyme specificity was set to Trypsin with up to 1 missed cleavage. Methyltiocysteine, 4-plex iTRAQ at N-terminus and Lys were set as fixed modifications and oxidation of methionine was selected as variable modification. False discovery rates (FDR) of 5% and 1% were calculated by Proteome Discoverer based on the search against the corresponding randomized database.

Results obtained from every LC-MS/MS analysis (from each SCX fraction, before and after the application of the static excluding list), were merged by Proteome Discoverer into a single file. A single list of globally identified proteins was obtained and peptides that could not be unequivocally attributed to a single protein were grouped into protein families to satisfy the principle of maximum parsimony. Data were filtered considering as positive hits all proteins identified with at least two unique peptides with medium confidence (FDR 5%) and quantified with at least 2 independent peptides.

Data obtained from the IEF fractionation were further elaborated. Theoretical values of pI peptides belonging to the same IEF fraction were calculated in batch using the “pI Calculator” software [11]. As explained elsewhere, we took advantage of the pI filtering application to optimize the cross correlation parameter (Xcorr) to assess the performance of different SEQUEST settings and consequently to select the best setting to use [7]. Using this approach, the best values for Xcorr were the following: Xcorr ≥1.7 for +2 tryptic peptides, ≥2.5 for +3 and +4 tryptic peptides for medium confidence identifications, Xcorr ≥1.6 for +2 tryptic peptides, ≥2.2 for +3 and +4 tryptic peptides for high confidence identifications. A ratio ≥1.5 or ≤ 0.66 among AdC and non-neoplastic PE samples was set as threshold for protein over-expression and under-expression, respectively.

### Immunohistochemistry

Immunohistochemistry was performed on 4–5 μm-thick formalin-fixed and paraffin-embedded (FFPE) sections from each tissue sample and cell-block. Staining was done automatically (BondmaX, Menarini, Florence, Italy), as described elsewhere [12,13], using the Bond Polymer Refine Detection kit (Leica Microsystem, Wetzlar, Germany), with anti-lumican antibody (polyclonal; Novus Biologicals, Littleton, USA; working dilution 1:100, 25 min, citrate buffer). Sections were then slightly counterstained with hematoxylin. Normal prostate sections were used as positive controls, whereas sections processed without primary antibody as negative ones. Lumican expression was jointly evaluated by two pathologists (R.C. and A.F.) unaware of any clinical information. Lumican expression was first assessed in normal lung tissue to understand its usual localization and intensity. Then it was semi-quantitatively scored on the base of the percentage of AdC cells with cytoplasmic immunoreation: 0 = no stain, 1 = 1–30%, 2 = 31–70%, and 3 = 71–100%. Finally, the area of stromal/extracellular lumican immunostaining was measured by superimposing a grid and counting the points intercepting the positive and negative zones as previously described [14]. The proportion of positive stromal/extracellular area was calculated, recorded as percentage, and classified as absent (<5%), low (5–50%), and high (>50%) expression.

### Cell lines

A549 lung cancer cells were purchased from the American Type Culture Collection (ATCC, Manassas, USA) and maintained in DMEM/F12 medium supplemented with 10% FBS and 1% antibiotic/antimycotic mixture. Fibroblasts were obtained from skin biopsies, as described elsewhere [15], and were cultured in HAM'S F-10 (Sigma-Aldrich, St. Louis, USA) supplemented with 10% FBS, 1 mmol/l glutamine, 100 U/ml penicillin, and 100 μg/ml streptomycin at 37°C until confluence. A549 cells and fibroblasts were cultured with serum-free medium for 24 h and then the quiescent medium was changed with DMEM supplemented with 1% Nutridoma mixture (Roche) for 48h before protein extraction.

### Immunoblotting

Cell pellets were homogenized in 300 μl M-PER Mammalian Protein extraction buffer (Thermo Scientific) using a MagNALyser Instrument (Roche). Culture medium (30 ml) from each cell line was collected, centrifuged for 30 min at 2,000 x g to pellet cell debris, and the proteins secreted in the supernatant were concentrated by acetone precipitation to 500 μl. Protein concentration was determined with microBCA (Thermo Scientific) method, using bovine serum albumin standard. Cleared protein lysates from the cell lines and the concentrated culture medium were separated on 10% SDS-PAGE gel and electro-transferred onto nitrocellulose membranes (Hybond ECL Amersham Biosciences, Glattbrugg, Switzerland). The membranes were blocked for 2 hrs using 5% BSA in 1X TBS and then incubated overnight with anti-lumican antibody (1:500 in dilution; Novus Biologicals). The immunoblotted proteins were incubated with horseradish proxidase-conjugated secondary antibodies (DAKO, Milan, IT) and visualized by a luminol-based chemiluminescence substrate (LumiGLO; KPL, Gaithersburg, USA). Images were processed by Molecular imager VersaDoc system (Biorad, Milan, Italy). The membranes were washed and reblotted with an anti GAPDH antibody used as positive control for intracellular proteins from A549 cancer cells and fibroblasts (Cell Signaling, Danvers, USA). All reactions were performed in triplicate.

### Statistical analysis

Correlations among IHC results and clinicopathological variables were analyzed by using the Kruskal-Wallis test and Wilcoxon rank sum test, as appropriate. A *p*-value <0.05 was considered statistically significant, while values in the range of 0.10≥ *p* ≥0.05 were assumed to indicate a statistical trend. All statistical analyses were performed using the R software (R Development Core Team, version 2.9; R Foundation for Statistical Computing, Vienna, Austria; www.R-project.org).

## Results

### Proteomic of pleural effusions

Overall, 109 protein groups were identified in both AdC and non-neoplastic PE samples (full list provided in S1 Table). Thirty proteins, mainly inflammatory or immune-related, were differentially expressed with an increased/decreased expression of at least 50% in AdC compared to non-neoplastic PE (Table 2). A protein overexpressed in neoplastic PE samples was selected for further validation: lumican. Lumican is a member of the small leucine-rich proteoglycan (SLRP) family involved in several cellular functions, including cell proliferation, migration, and differentiation [16–18].

**Table 2:** Proteomic analysis results.

Protein	Symbol	Ratio
*Overexpressed*		
G-protein coupled receptor 98	GPR98	2.546
N-acetylmuramoyl-L-alanine amidase	PGLYRP2	2.046
Monocyte differentiation antigen CD14	CD14	1.696
Complement component C7	C7	1.650
Isoform 2 of Sex hormone-binding globulin	SHBG	1.613
Ig gamma-2 chain C region	IGHG2	1.613
Alpha-2-HS-glycoprotein	AHSG	1.567
Retinol-binding protein 4	RBP4	1.544
Lumican	LUM	1.507
*Underexpressed*		
Protein S100-A8	S100A8	0.077
Isoform 2 of Rho GTPase-activating protein 25	ARHGAP25	0.131
Protein S100-A9	S100A9	0.155
Neutrophil defensin 1	DEFA1	0.192
Histone H2A type 1-H	HIST1H2AH	0.208
Isoform 2 of C-reactive protein	CRP	0.223
Histone H4	HIST1H4A	0.283
Histone H2B type 1-H	HIST1H2BH	0.283
Ig mu chain C region	IGHM	0.375
Isoform 2 of Brain acid soluble protein 1	BASP1	0.419
Actin	ACTB	0.444
Histone H3.3C	H3F3C	0.491
Ig kappa chain V-I region EU	-	0.492
Ig heavy chain V-III region TIL	-	0.548
Serum amyloid A protein	SAA1	0.554
Serum amyloid P-component	APCS	0.564
Ig gamma-1 chain C region	IGHG1	0.573
Isoform 2 of Arf-GAP with Rho-GAP domain, ANK repeat and PH domain-containing protein 1	ARAP1	0.578
Ig lambda chain V-I region WAH	-	0.587
Ig lambda-2 chain C regions	IGLC2	0.588
Ig kappa chain V-I region AG	-	0.590
Ig heavy chain V-I region HG3	-	0.593
Ig kappa chain V-IV region Len	-	0.601
Lipopolysaccharide-binding protein	LBP	0.604
Ig kappa chain V-III region WOL	-	0.618
Ig kappa chain V-II region TEW	-	0.643
Isoform 3 of 1-phosphatidylinositol-4,5-bisphosphate phosphodiesterase eta-1	PLCH1	0.649
Ig lambda chain V-III region SH	-	0.634
Ig kappa chain V-IV region JI	-	0.654

Proteins with different expression between lung adenocarcinoma and non-neoplastic pleural effusions.

### Immunohistochemistry

**Fig 1:**
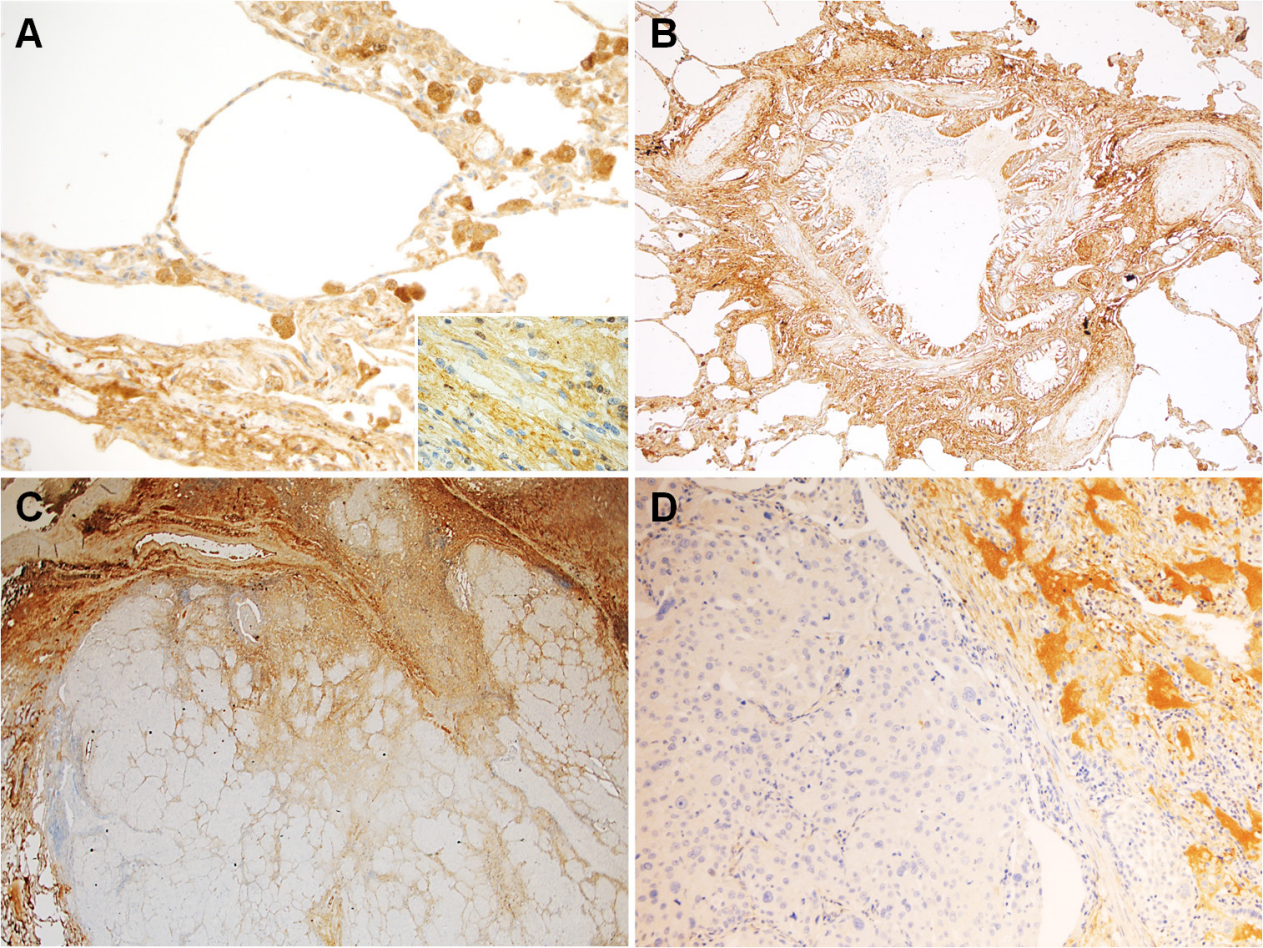
Representative images from the considered series. In normal lung (**A and B**), lumican immunoreaction was detectable not only in the cytoplasm of pneumocytes, columnar cells of the bronchi, fibroblasts (*inset*), and vascular smooth muscle cells, but also in collagen fibers of the perivascular and peribronchial connective tissues. In lung adenocarcinoma (**C and D**), lumican immunostaining was low in tumor cells and more diffuse in the surrounding tumor stroma and extracellular space. (Original magnification 200x in A and D, 100x in B, and 40x in C).

In normal lung tissue, lumican was expressed by both epithelial and mesenchymal cells. Indeed, a diffuse cytoplasmic immunoreaction was detectable in type I and II pneumocytes, columnar cells of the bronchi, fibroblasts and vascular smooth muscle cells (**Fig 1**). Moreover, lumican was detected in collagen fibers of the perivascular and peribronchial connective tissues (**Fig 1**).

**Fig 2:**
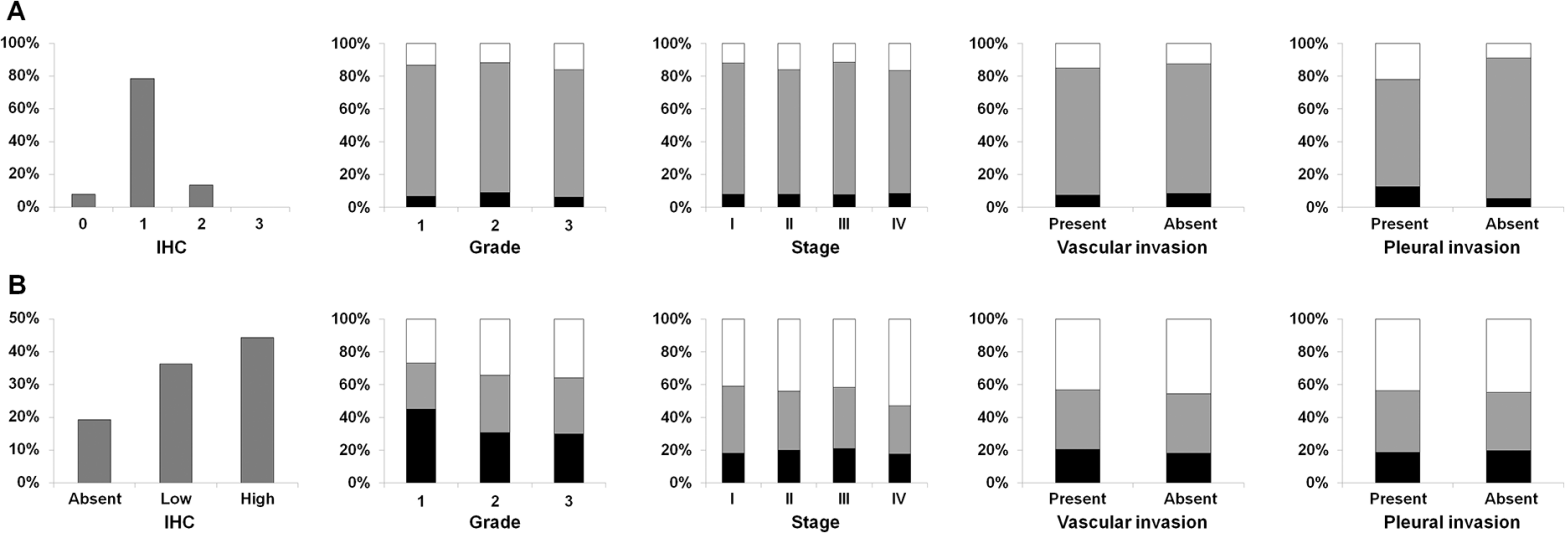
Immunohistochemistry results. Overall, grade, stage, vascular and pleural invasion distribution of immunohistochemical staining scores for lumican in lung adenocarcinoma (**A**) and in the surrounding stroma (**B**).

Overall, the IHC expression of lumican in the 88 resected AdCs was low. Indeed, none of the AdCs showed a lumican immunoreaction in more than 70% of tumor cells and in only 12 cases it exceeded the 31% (**Fig 2**). The large majority of AdCs (69 cases) was scored as 1 (1–30% of positive tumor cells) (**Fig 1**), whereas in 7 cases no immunostaining was detected (**Fig 2**). Any possible association of lumican IHC expression with tumor grade, stage (pTNM), and vascular/pleural invasion (evaluated on hematoxylin & eosin stained slides) was ruled out in resected AdCs. Lumican looked more expressed in the surrounding tumor stroma and extracellular space than in AdC cells and normal lung (**Fig 1**).

For this reason, stromal/extracellular lumican immunostaining was quantitatively measured in the connective tissue surrounding the tumors. In all cases, a stromal/extracellular positive immunoreaction was detected. However, stromal/extracellular positive lumican area resulted strong, uniform, and higher than 50% in 39 cases, whereas spanned the range of 5–50% in 32 cases. In the remaining 17 cases, immunoreaction was scarce and scattered (**Fig 2**). Again, lumican immunoreaction was not related to tumor grade, stage (pTNM), and vascular/pleural invasion.

**Fig 3:**
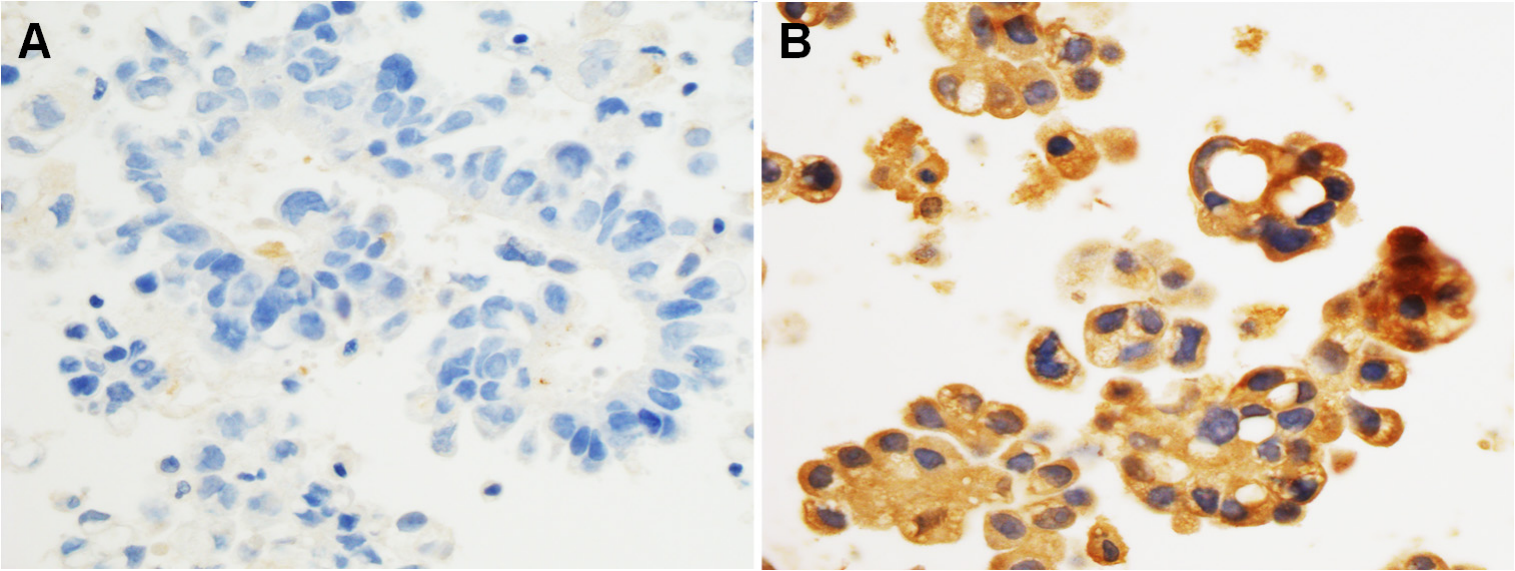
Representative images from the tumor cell-blocks. Lumican immunostaining was absent in lung adenocarcinoma cells (**A**), whereas it was strong and diffuse in breast carcinoma (invasive carcinoma of no special type) cells (**B**). (Original magnification 400x in A and B).

As for tumor cell-blocks, none of the 10 AdC as well as the 3 NSCLC-NOS ones showed lumican immunoreaction (**Fig 3**), in accordance with the low expression detected in the resected specimens. Contrariwise, the 5 breast carcinoma and 5 ovarian carcinoma specimens were all strongly positive for lumican (**Fig 3**).

### Immunoblotting

**Fig 4:**
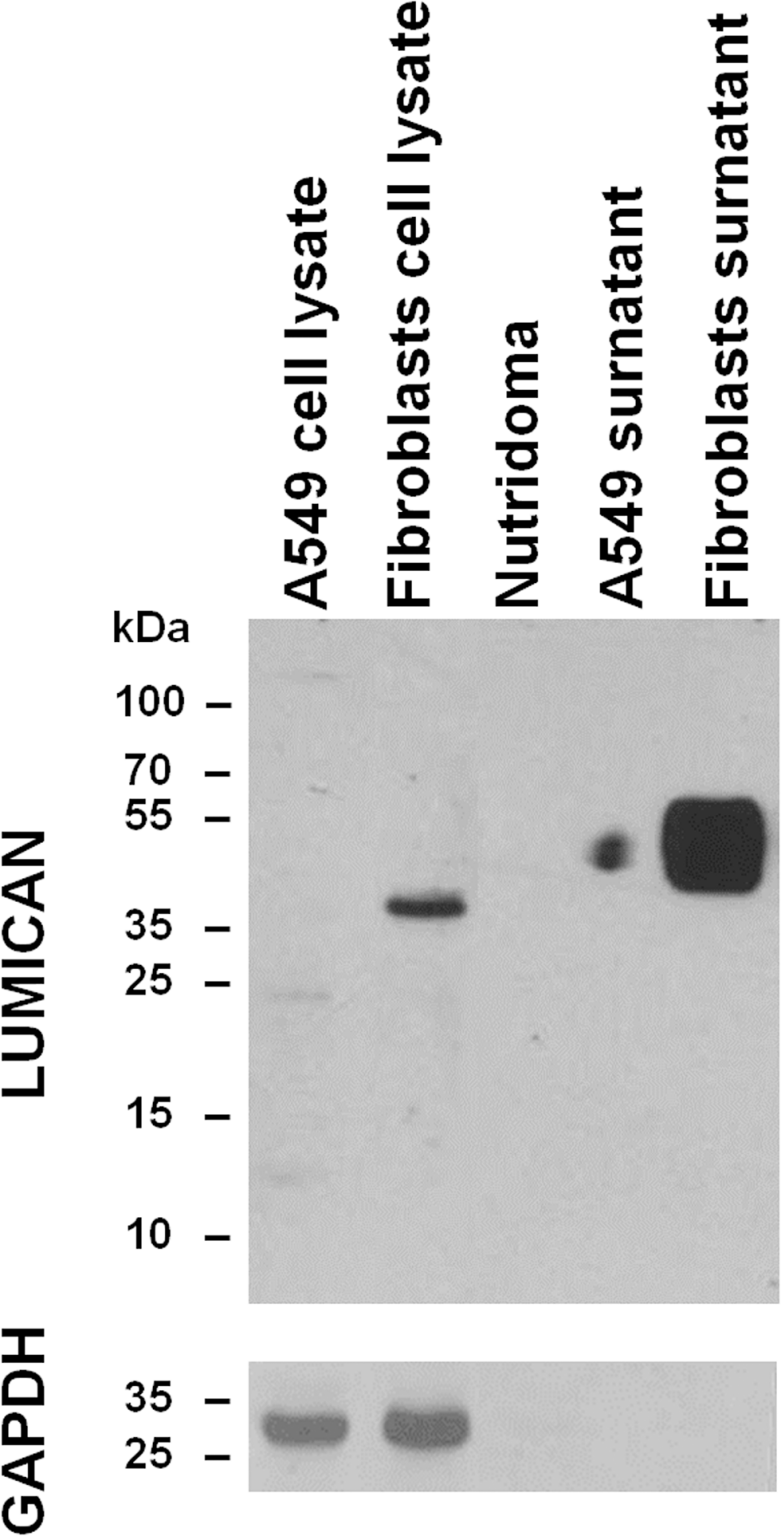
Immunoblotting results. Immunoblotting analysis of lumican in Nutridoma and both cell lysate and supernatant of A549 lung adenocarcinoma cell line and fibroblast culture.

In order to assess the source of lumican in PEs, immunoblotting was performed both in cell lysate and conditioned medium (a serum surrogate was used to avoid interferences) obtained from an AdC cell line (A549) and a fibroblast culture. Immunoblotting analysis showed that lumican was expressed in fibroblasts but not in AdC cells (**Fig 4**). As for the supernatant, lumican showed an uneven band in the A549 line and was clearly detected in the fibroblast one (**Fig 4**). The molecular weight of lumican in fibroblasts slightly exceeded 40 kDa, whereas in the corresponding conditioned medium ranged from 35 kDa to more than 55 kDa.

## Discussion

AdC is the most common histotype of lung cancer, the leading tumor worldwide for incidence and mortality. Despite the recent improvements in the treatment, prognosis remains poor mainly because a diagnosis is usually achieved in advanced stages, often with malignant PE. It is estimated that about 15% of patients with lung cancer have malignant PE [19]. Since AdC cells proliferate and secrete proteins in PE, a fluid with limited protein exchange with plasma, this may represent an effective source to discover novel biomarkers. In this study we applied the iTRAQ-LC-MS/MS proteomics approach to identify proteins with altered abundance in AdC PE as compared to non-neoplastic PE. Among the wide AdC proteome identified, in the present study we concentrated our attention on lumican and excluded by further investigations all the inflammatory or immune-related proteins. Since several stimuli can cause the elevation of this kind of proteins and many different diseases may share such alterations, they are hardly suitable to be employed as AdC specific biomarkers. However, also these proteins are interesting and deserve to be considered in future studies, as they may be useful in the prognostic stratification of patients accordingly to the most recent findings [20].

Lumican is a keratin sulphate belonging to the SLRP family of extracellular matrix (ECM) proteins and is expressed in different forms in several tissues and organs, such as cornea, bone, cartilage, artery, skin, kidney, and lung [17,18,21]. Physiologically, lumican is involved in the acquisition of corneal transparency, in the assembling of collagen fibrils, and in the regulation of pivotal biological processes, mainly angiogenesis, cell proliferation, migration, and adhesion [22–27]. These latter are important mechanisms also in cancer. Indeed, during tumor progression, cancer cells interact with the microenvironment and especially with ECM that not only provides a physical scaffold for cell adhesion and migration but also influences tumor angiogenesis [28,29]. Thus, alteration of lumican expression are supposed to be correlated to cancer spreading. Indeed, lumican has been reported to regulate cell migration in prostate and colorectal cancer, where is also related to a worse prognosis, and tumor progression in pancreatic cancer [17,18]. Notably, in breast cancer high stromal lumican expression is associated with high tumor grade and low estrogen receptor level [17,18]. Matsuda Y and colleagues studied lumican expression in a series of adenocarcinomas and squamous cells carcinomas of the lung by using IHC [30]. Accordingly with our findings, in the normal lung they observed lumican immunostaining in the bronchial epithelium, in vascular smooth muscle cells, and in fibroblasts and collagen fibers of the perivascular and peribronchial connective tissues [30]. However, Matsuda Y and colleagues did not detected lumican immunoreaction in the alveolar epithelium [30], whereas we highlighted lumican expression also in pneumocytes. Such incongruence may be explained by the different clones of the antibody employed. Indeed, we used a commercial polyclonal antibody recognizing regions in wide antigen aminoacidic sequence that may result in a broader positivity, whereas the antibody synthetized by Matsuda Y *et al* reacted against a polypeptide of only sixteen aminoacids. In their work, Matsuda Y and colleagues observed lumican immunostaining in both cancer cells and stromal tissues surrounding the tumor and found that tumor size and pleural invasion correlated with the expression levels of lumican in cancer cells [30]. Thus, the low lumican immunoreaction detected in the large majority of AdC cases in our series and the absence of any association of lumican expression with clinico-pathological variables were unexpected. Again, the clones of antibody may have affected the results. However, we did not assess lumican intensity but scored the percentage of positive tumor cells. On the other hand, the lack of lumican expression in AdC cell blocks corroborates its substantial absence also in the primitive site. The results of lumican IHC in AdC and non-AdC cell blocks suggest its possible utility as diagnostic biomarker and further studies should address this eventuality. Our findings agreed with those of Matsuda Y *et al* inasmuch as lumican expression was high in the stromal tissues surrounding the AdC cells and this did not correlate with clinic-pathological factors [30].

Immunoblotting results addressed that fibroblasts, rather than tumor cells, are a putative source for the high lumican levels recorded in AdC PEs. This finding, however, was in contrast to the data previously reported by Matsuda and colleagues. Indeed, they detected lumican protein in both cell extract and culture medium of the two AdC cell lines analyzed [30]. Again, the discrepancy could be due to the different antibodies utilized. Otherwise, the diverse culture conditions (we used Nutridoma) could be the explanation. A different glycosylation or a chemical digestion in culture medium could be the reason of the uneven band and the varied lumican molecular weights observed. It remains to be clarified if the high levels of lumican in PE may also due to release by the mesothelium lining the pleural cavity. Anyway, the lumican increase measured in PE could be detectable also in the plasma of AdC patients and its potential value as early diagnostic biomarker should be explored. The biological role of lumican in AdC is elusive and only functional studies will elucidate if it affects tumor progression. Indeed, understanding the molecular mechanisms through which AdC interacts with the microenvironment could provide the basis for the development of novel therapeutic modalities.

In conclusion, the present study demonstrated that: i) lumican is overexpressed in AdC PEs; ii) AdC cells in both resected specimens and cell blocks substantially lack lumican immunostaining; iii) about half of the considered AdC cases shows a strong stromal/extracellular lumican immunoreaction; iv) a presumed source of the high lumican levels detected in PEs could be represented by fibroblast. Further studies should address the diagnostic value of lumican and investigate its role in tumor progression.

## Supporting Information

S1 Table Proteomic analysis results. Full list of identified proteins in pleural effusions.
(DOCX)
