# Supplementary material for: Lumican Is Overexpressed in Lung Adenocarcinoma Pleural Effusions
Source: PLoS One. 2015 May 11;10(5):e0126458. doi: 10.1371/journal.pone.0126458 (PMC4427354; doi:10.1371/journal.pone.0126458)
Supplement: S1 Table — Full list of identified proteins in pleural effusions. (DOCX) [file pone.0126458.s001.docx]

**LIST OF PROTEINS**

| Ig kappa chain V-II region GM607 |
| --- |
| Protein S100-A8 |
| Isoform 2 of Rho GTPase-activating protein 25 |
| Protein S100-A9 |
| Neutrophil defensin 1 |
| Isoform 2 of C-reactive protein |
| Histone H4 |
| Histone H2A type 1-H |
| Histone H2B type 1-K |
| Ig mu chain C region |
| Isoform 2 of Brain acid soluble protein 1 |
| Actin, cytoplasmic 1 |
| Histone H3.3C |
| Serum amyloid P-component |
| Isoform 2 of Arf-GAP with Rho-GAP domain, ANK repeat and PH domain-containing protein 1 |
| Ig kappa chain V-IV region Len |
| Lipopolysaccharide-binding protein |
| Ig heavy chain V-I region HG3 |
| Ig lambda-2 chain C regions |
| Ig gamma-1 chain C region |
| Ig lambda chain V-I region WAH |
| Ig kappa chain V-II region TEW |
| Isoform 3 of 1-phosphatidylinositol-4,5-bisphosphate phosphodiesterase eta-1 |
| Ig kappa chain V-III region WOL |
| Serum amyloid A protein |
| Ig lambda chain V-III region SH |
| Ig kappa chain V-III region VG |
| Ig kappa chain V-I region EU |
| Ig delta chain C region |
| Ig alpha-1 chain C region |
| Ig kappa chain V-IV region JI |
| Ig heavy chain V-III region TIL |
| Ig heavy chain V-III region BRO |
| Isoform 3 of Protein AHNAK2 |
| Ig kappa chain V-I region AG |
| CD5 antigen-like |
| Ig gamma-3 chain C region |
| Actin-related protein 10 |
| Thyroxine-binding globulin |
| Fibulin-1 |
| Ig heavy chain V-III region GAL |
| Ankyrin-3 |
| Complement factor H-related protein 1 |
| Plasminogen |
| Alpha-1-acid glycoprotein 2 |
| Haptoglobin |
| Pigment epithelium-derived factor |
| Complement factor H |
| Ig kappa chain C region |
| Complement C5 |
| Hemoglobin subunit beta |
| Isoform 2 of NEDD8 ultimate buster 1 |
| Isoform 2 of Inter-alpha-trypsin inhibitor heavy chain H4 |
| Serum amyloid A-4 protein |
| Isoform 5 of Myosin-VI |
| Alpha-2-macroglobulin |
| Apolipoprotein M |
| Isoform 4 of Titin |
| Isoform 2 of Phospholipid transfer protein |
| Monocyte differentiation antigen CD14 |
| Corticosteroid-binding globulin |
| Complement factor I |
| Isoform 4 of Fibronectin |
| Alpha-2-antiplasmin |
| Isoform 2 of Gelsolin |
| C4b-binding protein alpha chain |
| Complement component C6 |
| Complement C4-A |
| Alpha-1-acid glycoprotein 1 |
| Vitronectin |
| Alpha-1-antichymotrypsin |
| Complement factor B |
| Leucine-rich alpha-2-glycoprotein |
| Kallistatin |
| Isoform 4 of Clusterin |
| Complement C2 |
| Apolipoprotein D |
| Vitamin K-dependent protein S |
| Immunoglobulin lambda-like polypeptide 5 |
| Isoform 2 of DNA-dependent protein kinase catalytic subunit |
| Apolipoprotein B-100 |
| Heparin cofactor 2 |
| Complement component C9 |
| Immunoglobulin J chain |
| Alpha-1-antitrypsin |
| Apolipoprotein A-IV |
| Coagulation factor XII |
| Isoform 2 of TBC1 domain family member 4 |
| Isoform 5 of Myosin-VIIa |
| Isoform LMW of Kininogen-1 |
| Histidine-rich glycoprotein |
| Complement C1r subcomponent |
| Ceruloplasmin |
| Complement C3 |
| Complement component C7 |
| Tankyrase-2 |
| Isoform E of Proteoglycan 4 |
| Complement C1s subcomponent |
| Serum paraoxonase/arylesterase 1 |
| tRNA (cytosine(34)-C(5))-methyltransferase |
| Apolipoprotein C-III |
| Isoform 4 of Kinesin-like protein KIF20B |
| Isoform 5 of Regulating synaptic membrane exocytosis protein 1 |
| Isoform 6 of Uncharacterized protein KIAA1109 |
| Angiotensinogen |
| Inter-alpha-trypsin inhibitor heavy chain H1 |
| Isoform Beta-2 of Serine/threonine-protein phosphatase 2A 56 kDa regulatory subunit beta isoform |
| Plasma protease C1 inhibitor |
| Ig lambda chain V-III region LOI |
| Zinc-alpha-2-glycoprotein |
| Fibrinogen beta chain |
| Afamin |
| Apolipoprotein E |
| Ig gamma-4 chain C region |
| Apolipoprotein L1 |
| Histone H1x |
| Serum albumin |
| Isoform Gamma-A of Fibrinogen gamma chain |
| Alpha-1B-glycoprotein |
| Apolipoprotein A-I |
| Protein AMBP |
| Ig lambda-7 chain C region |
| Isoform 3 of NADPH oxidase activator 1 |
| Prothrombin |
| Apolipoprotein A-II |
| Vitamin D-binding protein |
| Isoform 2 of Fibrinogen alpha chain |
| Inter-alpha-trypsin inhibitor heavy chain H2 |
| Apolipoprotein C-II |
| Hemopexin |
| Antithrombin-III |
| Isoform 2 of Sex hormone-binding globulin |
| Apolipoprotein C-I |
| Serotransferrin |
| Retinol-binding protein 4 |
| Alpha-2-HS-glycoprotein |
| Lumican |
| Ig gamma-2 chain C region |
| Beta-2-glycoprotein 1 |
| N-acetylmuramoyl-L-alanine amidase |
| G-protein coupled receptor 98 |
